# The Kilogram and Measurements of Mass and Force

**DOI:** 10.6028/jres.106.003

**Published:** 2001-02-01

**Authors:** Z. J. Jabbour, S. L. Yaniv

**Affiliations:** National Institute of Standards and Technology, Gaithersburg, MD 20899-0001

**Keywords:** force, kilogram, mass, uncertainty

## Abstract

This paper describes the facilities, measurement capabilities, and ongoing research activities in the areas of mass and force at the National Institute of Standards and Technology (NIST). The first section of the paper is devoted to mass metrology and starts with a brief historical perspective on the developments that led to the current definition of the kilogram. An overview of mass measurement procedures is given with a brief discussion of current research on alternative materials for mass standards and surface profiles of the U.S. national prototype kilograms. A brief outlook into the future possible redefinition of the unit of mass based on fundamental principles is included. The second part of this paper focuses on the unit of force and describes the realization of the unit, measurement procedures, uncertainty in the realized force, facilities, and current efforts aimed at the realization of small forces.

## 1. The Kilogram and Mass Measurements

### 1.1 The Unit of Mass

From the early history of humankind to modern times, mass measurements have formed the corner stone for trade and commerce. The use of weights and balances as tools to perform mass measurements for trade dates back thousands of years and is most likely associated with the early civilizations of the Nile Valley and the Middle East. Since those times, mass standards and the technology of balances and mass measurements have greatly evolved to meet the growing and changing needs of society. The activities of everyday life have always been affected either directly or indirectly by mass measurements. Whenever one buys groceries, takes medication, designs a bridge, space shuttle, or airplane, trades goods—whether grains, gold, or gemstones—mass plays a crucial and vital role. In addition to the direct impact on trade and commerce, mass measurements impact the scientific community as well as a broad range of manufacturing industries including aerospace, aircraft, automotive, chemical, semiconductor, materials, nuclear, pharmaceutical, construction, and instrument manufacturing. To ensure equity and equivalence in trade and manufacturing at the national and international levels, uniform standards are needed. While mass standards have been in existence for thousands of years and some countries had rather controlled policies on weights, uniformity was not guaranteed across boundaries and sometimes not even within the boundaries of one country. In the United States, the unit of mass was the avoirdupois pound, and many standards were brought over from England to the colonies to serve as standards for trade. However, this did not form a robust system and non-uniformity remained a major issue. The United States government formally recognized this need and empowered Congress to “fix the standards of weights and measures” in the Constitution of the United States. Many attempts at adopting a uniform system of weights were made. It wasn’t until 1875 that the United States along with 16 other countries signed the Meter Convention that established the foundations of the International System of Units (SI) that would finally provide the long sought after uniformity in the standards of weights and measures. A detailed account of the history of weights and measures in the United States can be found in Ref. [1].

The foundation of the SI lies with the 1791 decision of the French National Assembly to adopt a uniform system based entirely on the unit of length, the meter, defined at the time as being equal to one ten-millionth of the length of the quadrant of the earth meridian. The unit of mass would be the mass of a cubic decimeter of water at 4 °C, the temperature of maximum density. Based on these definitions, a prototype meter and kilogram were manufactured and deposited in the Archives of the French Republic in 1799 forming the basis of the presently adopted SI. The prototype kilogram became known as the Kilogram of the Archives. In 1875, the Meter Convention founded the “Comité International des Poids et Mesures” (CIPM), which took the responsibility of manufacturing replicas of the meter and kilogram prototypes, and the “Bureau International des Poids et Mesures” (BIPM) whose function would be to serve as the custodian of the prototypes, carry out future international comparisons, and serve as the center for disseminating the metric system. In 1878, three 1 kg cylinders, KI, KII, and KIII, made of 90 % platinum—10 % iridium alloy were ordered from Johnson Matthey in England; they were delivered in 1879. They were polished, adjusted, and compared with the Kilogram of the Archives by four observers in 1880 at the Observatory of Paris. The mass of KIII was found to be the closest to that of the Kilogram of the Archives. KIII was placed in a safe at the BIPM in 1882, was chosen by the CIPM to be the International Prototype Kilogram, and was ratified as such by the 1st “Conference Generale des Poids et Mesures” (CGPM) in 1889. In 1901, the 3rd CGPM in Paris established the definition of the unit of mass: “The Kilogram is the unit of mass; it is equal to the mass of the International Prototype of the Kilogram.” The International Prototype Kilogram is often referred to as “IPK” and is frequently designated with the Gothic letter K. In 1884, 40 replicas of the kilogram were delivered from Johnson Matthey; they were compared to the mass of the IPK in 1888. In 1889, 34 of these replicas were distributed to the signatories of the Meter Convention who requested them. Calibration certificates accompanied the replicas with mass values based on comparisons with the IPK. These replicas were in turn used by the different countries as national standards. At that time, the United States was allocated two Pt-Ir prototype kilograms, K20 and K4. K20 arrived in the United States in 1890 and was designated as the primary national standard of mass. K4 arrived later that same year and was assigned as a check standard to monitor the constancy of K20. Over a century later, K20 and K4 still hold their respective positions. The six remaining replicas were kept at the BIPM to serve as check standards for IPK. In addition to the original 40 copies, more replicas were constructed to serve the growing needs of the international community. In 1996, the U.S. acquired a new prototype kilogram, K79.

Since its foundation in 1875 and until 1973, the BIPM used two equal-arm mechanical balances: the Bunge balance that was in service between 1879 and 1951 and the Rueprecht balance that served the BIPM’s needs from 1878 until 1974 [2]. In 1970, the National Bureau of Standards (NBS), predecessor to NIST, donated a 1 kg balance, known as NBS-2, to the BIPM. NBS-2 was designed and developed at NIST to allow for the simultaneous measurement of six 1 kg standards. The unique constant-load, double-knife-edge design allowed metrologists to achieve state-of-the-art resolution and repeatability [3]. NBS-2 was used for the calibration of 1 kg standards at the BIPM between 1973 and 1992, replacing the Rueprecht balance that was nearing 100 years of age. Currently the BIPM uses state-of-the-art balances that are either commercially available or developed at the BIPM.

The unit of mass is only available at the BIPM. Therefore, the prototypes serving as national standards of mass must be returned periodically to the BIPM for calibration either on an individual basis, which could be done anytime, or as part of a simultaneous recalibration of all the prototypes known as “periodic verification.” Since the existence of the prototypes there has been only three such periodic verifications. The latest one, the third periodic verification, took place between 1988 and 1992. For it, the IPK was used with the NBS-2 balance. The results of the third periodic verification demonstrated a long-term instability of the unit of mass on the order of approximately 30 μg/kg over the last century [4]; this instability is attributed to surface effects that are not yet fully understood. Mass standards, including IPK and its replicas, are stored in ambient air; therefore, their surfaces are subject to the adsorption or absorption of atmospheric contamination resulting in a gain in mass over time; they also may lose mass from usage. The BIPM has developed a recommended method [5] for cleaning platinum-iridium (Pt-Ir) prototypes to remove surface contaminants and restore the artifact to its original state. In 1989, the CIPM interpreted the 1901 definition of the kilogram [6]. The interpretation, which does not imply a redefinition of the kilogram, refers to the kilogram as being equal to the mass of the IPK just after cleaning and washing using the BIPM method.

In 2001, the kilogram remains as the only SI base unit defined by an artifact and thus is constantly in danger of being damaged or destroyed. In addition, the definition of the kilogram makes no provision for either the artifact surface parameters or for any environmental storing conditions. Environmental effects combined with wear and other material and surface properties constitute the most probable reason for the observed instability in mass over time. The instability in the definition of the kilogram propagates to other SI base units that are tied to the kilogram such as the ampere, mole, and candela. It also propagates to derived quantities such as density, force, and pressure. Therefore, the impact of the instability in the unit of mass spans a broad range of applications in the scientific and engineering sectors.

While comparisons of nearly identical 1 kg mass standards can be performed with a relative precision of 10^−10^ with commercially available balances and with 10^−12^ with special balances, it is clear that the limitation in the field of mass metrology lies within the artifact definition itself. Therefore, the ultimate need for mass metrology is to redefine the unit of mass in terms of a fundamental constant of nature. At the same time, it is also crucial to pursue more stable and ideal artifacts and transfer standards, as this will be, at least for the foreseeable future, the only practical dissemination tool.

### 1.2 Mass Measurement

#### 1.2.1 Cleaning and Handling of Mass Standards

Mass standards are typically stored and used in ambient air; therefore, they accumulate contaminants and must be cleaned occasionally to restore them to their original mass values. Cleaning policies and protocols depend on the artifact material and can vary greatly among laboratories.

The internationally accepted cleaning method of the platinum-iridium prototypes is known as “the BIPM cleaning method” and it is described in Ref. [5]. This method was developed at the BIPM between 1939 and 1946; it evolved from years of experimentation on cleaning methods that included using a variety of solvents. The currently used BIPM method consists of rubbing the artifact with chamois cloth that has been soaked in a mixture of equal proportions of ether and alcohol. Since the ether and alcohol mixture leaves a residue, the artifact is then cleaned in a jet of steam from doubly distilled water. Results show that this procedure is effective in removing contamination from the surface [4]. It is worth noting that this method relies on the human touch and therefore can be highly irreproducible. NIST follows this protocol to clean the national standards of mass K20, K4, and K79 when necessary. All other NIST mass standards and those submitted for calibration are generally made of stainless steel and are subjected to different cleaning procedures, depending on their size and construction, as described below.

Mass standards made of one-piece construction in the range of 1 g to 1 kg are cleaned by washing the artifacts with condensing alcohol vapor, usually referred to as “vapor degreasing.” Following washing, the artifacts are allowed to dry and any droplets on the surface are gently patted dry.

Mass standards larger than 1 kg and all weights of two-piece construction are cleaned by wiping with lint-free cheesecloth moistened with alcohol.

Fractional weights (1 mg to 500 mg) are cleaned by soaking them in alcohol followed by gently patting them dry.

When mass standards are contaminated with oily residues, they are cleaned with acetone followed by alcohol using lint-free cheesecloth. Typically, unless specified otherwise by the customers, all mass standards are cleaned before calibration.

After cleaning, weights are allowed to stabilize for a period of 7 to 10 days before calibration. The stabilization period is determined based on the results of characterization of the stability of mass standards by monitoring the mass of a selected set of weights after cleaning [7]. Before calibration, weights are stored inside or near the balance, under cover, for a period of at least 24 hours to reach thermal equilibrium with the surrounding temperature. Weights larger than 10 kg require a longer thermal stabilization period depending on their size.

The handling of mass standards requires special precautions. Care must always be taken to minimize the risks of dropping and therefore damaging the surface of the artifacts. In order to minimize contamination, mass standards must always be kept in a relatively dust free environment with appropriate air filtration. When not in use, mass standards must be kept under a glass bell jar or other appropriate cover. In addition, mass standards must never be handled with bare hands. Usually special handling devices such as tweezers are used to avoid direct contact. If handling by hand is required, gloves must be worn. Gloves must be chosen to be powder free and such that their use doesn’t result in contamination of the artifact. In addition to contamination, handling by direct contact with the human body results in change in temperature that will later require additional thermal stabilization time. If handling devices are used, the part that comes in contact with the mass standards must be clean, non-abrasive, and non-magnetic. Before calibration, dust particles that could have accumulated on the surface of the artifacts can be removed by either blowing air using a bulb type rubber syringe or by lightly brushing with a clean brush.

#### 1.2.2 Density Determination

High precision mass measurements require applying an air buoyancy correction that in turn requires the knowledge of the air density as well as the volumes or densities of the artifacts.

The air density is computed using the internationally accepted equation for the determination of the density of moist air [8] from the measurement of the CO_2_ con-concentration, temperature, barometric pressure, and relative humidity. All the environmental transducers are regularly calibrated by the appropriate groups at NIST and are traceable to the national standards of temperature, pressure, and humidity. The standard uncertainty in the air density is 0.000 17 kg/m^3^ based upon standard uncertainties of the measured temperature, barometric pressure, and relative humidity of 5 mK, 10 Pa, and 0.5 %, respectively.

Mass standards typically have rounded edges, knobs, and recessed bottoms, therefore determining the volume by geometric means is neither very accurate nor practical. Volumes (or densities) are measured using an immersed balance and hydrostatic weighing systems. Since both systems require immersion of the weights in a fluid, all standards must be of a one-piece construction to avoid introducing fluid into any cavities in the weights.

The immersed balance procedure developed at NIST by Davis and Schoonover [9] uses the novel idea of immersing a modified electronic balance in a bath of fluorocarbon fluid. Volumes of mass standards in the range from 100 g to 1 kg are measured by comparison to volume standards, of the same nominal value, determined to a higher precision by the hydrostatic technique described below. Check standards are incorporated in the measurements to monitor the accuracy of the process. The relative combined standard uncertainty in the density using this procedure is 0.004 %. Artifacts of other denominations between 100 g and 1 kg, and special requests requiring higher precision, are performed using hydrostatic weighing techniques.

The hydrostatic weighing procedure uses silicon as density reference standards [10]. The use of solid objects as reference standards for density measurements was first developed at NIST in 1974 [11]; this method eliminated the use of water as a density reference standard and is currently used in most laboratories where high-precision density measurements are required. The hydrostatic weighing system currently in use is essentially the same system developed at NIST in 1974 for the measurement of silicon density standards with an electronic top-loading balance replacing the mechanical balance. A fluorocarbon fluid is used for most of the measurements while water is occasionally used. Check standards are incorporated in the measurements to monitor the accuracy of the process. Mass determinations in air and in the fluid are done against NIST mass standards to eliminate errors due to nonlinearity of the balance. The density of the silicon reference standards used is known with a relative standard uncertainty of 7.5×10^−7^ from hydrostatic weighing by comparison against stainless steel spheres whose volumes were measured using laser interferometry [1]. The relative combined standard uncertainty in the density using the hydrostatic system is 0.001 %.

Typically for large weights (above 1 kg), the density of a sample of the same material is measured. The sample should preferably be from the same bar and cut from a location as close to the weight as possible to minimize any effects due to nonhomogeneity of the material.

For weights smaller than 100 g and for all weights made of two-piece construction, either the manufacturer’s stated density or the density supplied by the customer is used.

A new, fully automated hydrostatic density measuring system based on silicon spheres as reference standards is currently being developed. A new system for measuring the density of artifacts in the range from 2 kg to 10 kg is also under development. Both systems are expected to be in operation by 2002.

### 1.3 Dissemination of the Unit of Mass

**Fig. 1:**
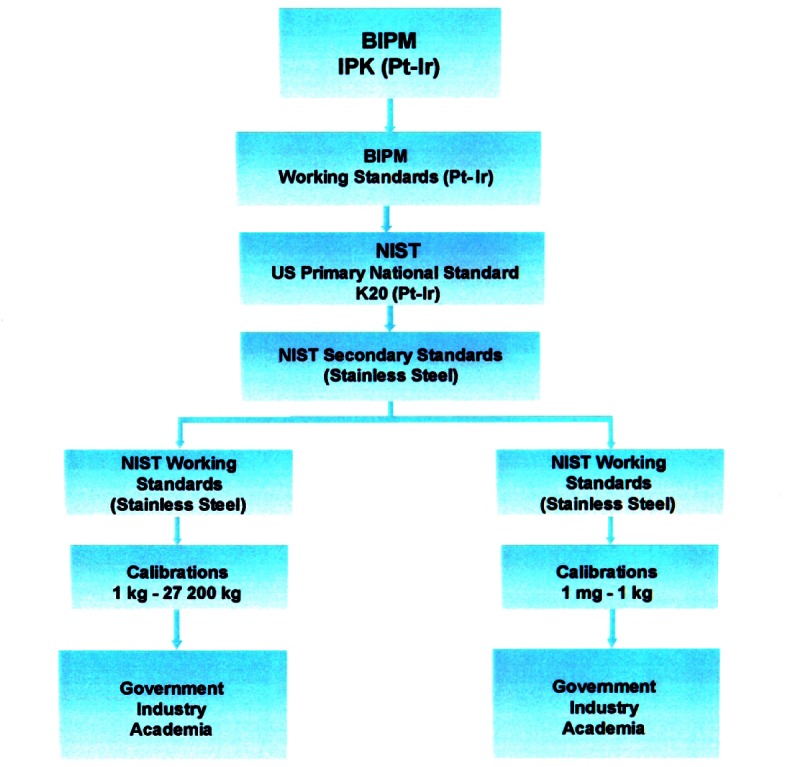
Traceability of mass measurements to the SI unit of mass.

While the unit of mass is defined at the one kilogram level, the mass scale must be realized over a range broad enough to be of practical use in commerce and manufacturing. The first stage in the realization of the mass scale is to disseminate the unit from the International Prototype Kilogram to the national standard followed by a transfer to a set of working standards at the one kilogram level. This is followed by dissemination to multiples and submultiples of the kilogram covering the range from 1 mg to 27 200 kg. The traceability from the International Prototype Kilogram to the multiples and submultiples of the kilogram is shown in Fig. 1. The procedures involved are discussed in the following sections.

#### 1.3.1 Dissemination From the International Prototype Kilogram to the National Standards

The link between the SI unit of mass and the U.S. national standard of mass is maintained through periodic calibrations of the national standard at the BIPM. The U.S. national standard of mass, K20, was calibrated at the BIPM six times during its lifetime, the latest calibration being in 1999 when it was calibrated against the BIPM working standards using a commercial electronic balance. K4, the U.S. check standard, was calibrated at the BIPM three times. Table 1 shows the dates of calibration along with the masses reported on the calibration certificates of the U.S. prototypes from the BIPM. The combined standard uncertainty (coverage factor *k* = 1) ranged from 2 μg to 4 μg. The densities of the prototype kilograms K20 and K4 have been measured at the BIPM using hydrostatic weighing techniques with water as a reference standard; the measured values are 21 539.14 kg/m^3^ and 21 531.77 kg/m^3^, respectively with a relative standard uncertainty estimated at 0.003 %[13].

The masses reported in Table 1 are obtained after cleaning and washing of the prototypes using the BIPM method. When the prototype kilograms are not cleaned, a correction to the “after cleaning” mass is applied. This correction is based on a model developed by the BIPM. Based on this model, a platinum-iridium kilogram gains 1.11 μg per month for the first 3 months after cleaning. The rate of change of mass then decreases to approximately 1 μg per year [4].

#### 1.3.2 Dissemination to the Stainless Steel Secondary Standards

The U.S. unit of mass is traceable to the IPK through the primary national standard of mass, K20. The mass unit is first transferred from K20 to a set of secondary stainless steel (SS) kilogram standards manufactured from nonmagnetic SS alloys with nominal density of 8000 kg/m^3^, polished surfaces, and chamfered edges. Prior to the mass calibration, the densities are determined using the hydrostatic weighing method with silicon reference standards, as described above.

**Fig. 2:**
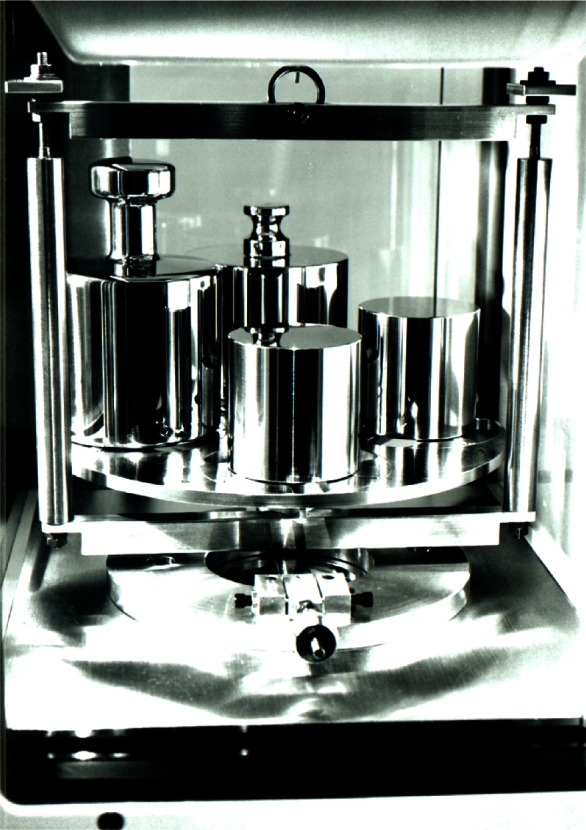
The national standard of mass K20, K4, and secondary stainless steel standards inside the balance during measurement.

The standards are cleaned after the density measurements by vapor degreasing and are allowed to stabilize before calibration as outlined earlier. Subsequent cleaning is performed only if a weight has been subject to unusual contamination. A commercially available and fully automated electronic 1 kg mass comparator with a resolution of 1 μg is used. This comparator is equipped with a weight-handling mechanism that allows for the simultaneous measurement of four mass standards of equal nominal mass, which in this case is 1 kg. Figure 2 shows K20, K4, and two stainless steel kilogram standards inside the balance during calibration. K20 and K4 are cylindrical weights while the knob weights are the stainless steel secondary standards. Since a balance is essentially a force transducer that measures the net vertical forces acting on an object, the balance reading reflects the difference between the gravitational and buoyant forces; if the balance is calibrated and the sensitivity is measured [14], the balance reading allows for the determination of the mass value. Typically, mass measurements are performed by comparison weighing involving a reference R and an unknown X:

$$m_{R}-\rho_{a}V_{R}=C_{R}$$ (1)

$$m_{X}-\rho_{a}V_{X}=C_{X}$$ (2)

where *m*_R_ and *m*_x_, *V*_R_ and *V*_x_, *C*_R_ and *C*_x_ denote the mass, volume, and balance reading for the reference R and the unknown X, respectively while *ρ*_a_ refers to the air density during the measurement.

Comparing the above two equations by taking the difference allows for the determination of the value of the unknown:

$$m_{X}=m_{R}-\rho_{a}\left(V_{R}-V_{X}\right)-C$$ (3)

where *C* = *C*_R_−*C*_X_, and the assumption was made that the air density *ρ*_a_ does not change during this comparison. Equation (3) represents the simplest and most fundamental mass measurement process. It is evident from Eq. (3) that the air buoyancy correction is proportional to the difference in volumes between the reference and the unknown. Therefore, the comparison of two artifacts of different volumes such as a 1 kg weight made of Pt-Ir and a 1 kg weight made of stainless steel results in a buoyancy correction of 94.2 mg assuming an air density of 1.2 kg/m^3^, a volume of 125 cm^3^ for a stainless steel kilogram, and a volume of 46.5 cm^3^ for a Pt-Ir kilogram. In order to minimize any effect of balance nonlinearity, small weights with total mass of approximately 94 mg are added to the stainless steel kilograms. The stainless steel kilograms are calibrated in pairs, denoted X1 and X2, against the national standard K20 while K4 acts as a check standard. The small added masses to X1 and X2 are represented by z1 and z2, respectively. Difference measurements *Y_i_* are obtained with all possible combinations between all four standards; this results in six differences:

**Table:** 

Observation	(1)	(2)	(3)	(4)
	K20	K4	X1+z1	X2+z2
*Y*_1_	+	−		
*Y*_2_	+		−	
*Y*_3_	+			−
*Y*_4_		+	−	
*Y*_5_		+		−
*Y*_6_			+	−

The (+) and (−) signs in the above matrix indicate the order in the difference measurement: (+) and (−) for observation *Y*_1_ indicates a measurement of the difference between K20 and K4 where K20 is measured first. Therefore, the above matrix translates into the following equations after taking into account the buoyancy correction:

$$\left(m_{K20}-\rho_{a1}V_{K20}\right)-\left(m_{K4}-\rho_{a1}V_{K4}\right)=Y_{1}$$ (1)

$$\left(m_{K20}-\rho_{a2}V_{K20}\right)-\left(m_{X1}-\rho_{a2}V_{X1}+m_{z1}-\rho_{a2}V_{z1}\right)=Y_{2}$$ (2)

$$\left(m_{K20}-\rho_{a3}-V_{K20}\right)-\left(m_{X2}-\rho_{a3}V_{X2}+m_{z2}-\rho_{a3}V_{z2}\right)=Y_{3}$$ (3)

$$\left(m_{K4}-\rho_{a4}-V_{K4}\right)-\left(m_{X1}-\rho_{a4}V_{X1}+m_{z1}-\rho_{a4}V_{z1}\right)=Y_{4}$$ (4)

$$\left(m_{K4}-\rho_{a5}-V_{K4}\right)-\left(m_{X2}-\rho_{a5}V_{X2}+m_{z2}-\rho_{a5}V_{z2}\right)=Y_{5}$$ (5)

$$\left(m_{X1}-\rho_{a6}V_{X1}+m_{z1}-\rho_{a6}V_{z1}\right)-\left(m_{X2}-\rho_{a6}V_{X2}+m_{z2}-\rho_{a6}V_{z2}\right)=Y_{6}$$ (6)

Such a series of difference measurements is known as a weighing design. This particular weighing design is referred to as a 4-1 design indicating that it involves four weights of equal nominal mass. Fixing the value of one of the standards allows one to solve this system of equations using the method of the least squares [15]. In this case, the mass of K20 is known from the calibration at the BIPM and is therefore used as the restraint:

$$m_{K20}=R.$$ (7)

These weighing designs have been developed at NIST by Cameron et al. in 1979. A full description can be found in Ref. [15]. Such measurements allow one to determine the masses of the unknown standards X1 and X2 as well as K4 from linear combinations of the mass differences *Y*_1_, ……..,*Y*_6_ and the value of the restraint as described in Ref. [15] after correcting each mass difference for the buoyancy correction associated with the standards involved [16].

Since the mass of K4 is known from a calibration at the BIPM, the determination of its mass here serves as a check of the accuracy of the process as discussed below.

The difference in the geometry between the Pt-Ir and stainless steel standards results in a difference in the relative locations of the center of mass. This results in a change in the measured mass that is proportional to the gravitational gradient over the range between the locations of the two centers of gravity. The gravitational correction is given by:

$$1\;\text{kg}\frac{1}{g}\frac{\partial g}{\partial h}\left(\Delta h\right)$$ (8)

where Δ*h* represents the distance between the centers of mass of the two artifacts being compared, *g* is the acceleration of free fall, and $\frac{\partial g}{\partial h}$ is the gravitational field gradient. In order to quantify this correction, the gravitational gradients as well as the absolute acceleration of free fall at the location where the mass calibrations are performed were measured by the National Geodetic Survey to be [(3.35×10^−6^)±(0.06×10^−6^)] s^−2^ and (9.800 998 6±10^−7^) m/s^2^, respectively. For Δ*h* = 1 cm, which is typical, the gravitational correction is 3 μg.

The combined standard uncertainty in the mass of a secondary stainless steel kilogram is computed from the basic equation for mass determination [Eq. (3)] based on the ISO Guide for the Expression of Uncertainty in Measurement [17], resulting in the following contributions:

- Air density: the uncertainty component due to air density is proportional to the difference in volume between the two standards being compared. It is evident here that the dominant component is due to the large difference in volume ≈80 cm^3^) between the Pt-Ir and secondary SS kilograms. This uncertainty component is *u*_air_ = 13.3 μg for an uncertainty in the air density of 0.000 17 kg/m^3^.
- Balance: the uncertainties due to repeatability and reproducibility are computed in accordance with the model developed by C. M. Croarkin using the procedures outlined in Ref. [18]. In this case, *u*_balance_ = 2.3 μg.
- Reference, K20: this component is taken from the calibration certificate of K20 supplied by the BIPM; *u*_reference_ = 4 μg based on the 1999 calibration certificate.
- Added masses: uncertainty in the small masses added to the stainless steel kilograms to compensate for the large difference due to the buoyancy correction. The uncertainty in the 94 mg as obtained from previous calibration against NIST standards is given by *u*_add-mass_ = 0.1 μg.
- Volume of standards: this component of the uncertainty, *u*_volumes_, is due to the uncertainty in the volumes of the reference K20 and the unknown weights, X1 or X2. This uncertainty component is negligible when the air densities at the time of calibration and the time of use of the standards are comparable [19].
- Other less significant uncertainty components not included in the above list are: *u*_temperature_, due to possible errors in the temperature volume expansion coefficients and *u*_gravity_, from the gravitational corrections.

The combined standard uncertainty is given by

$$U=\sqrt{u_{\text{air}}^{2}+u_{\text{balance}}^{2}+u_{\text{reference}}^{2}+u_{\text{add-mass}}^{2}+u_{\text{volumes}}^{2}+u_{\text{temperature}}^{2}+u_{\text{gravity}}^{2}}.$$ (9)

When all the uncertainties mentioned above are included, the combined standard uncertainty of the mass of a secondary stainless steel standard kilogram is found to be 14 μg (coverage factor *k* = 1).

The secondary standards are used as reference standards in the calibration of the working standards at the 1 kg level. The calibration procedure is similar and uses the same automated comparator. However, since the secondary and working standards have similar volumes, the buoyancy correction is very small. Therefore, the need for added masses is eliminated and the uncertainty in the buoyancy correction is minimized. The major contributions to the uncertainty become the uncertainty in the reference standard used, *u*_reference_ = 14 μg and the combined repeatability and reproducibility of the balance, *u*_balance_ = 2.3 μg. The combined standard uncertainty in the 1 kg working standard is therefore computed to be 14.2 μg.

#### 1.3.3 Dissemination to Multiples and Submultiples of the Kilogram

**Fig. 3:**
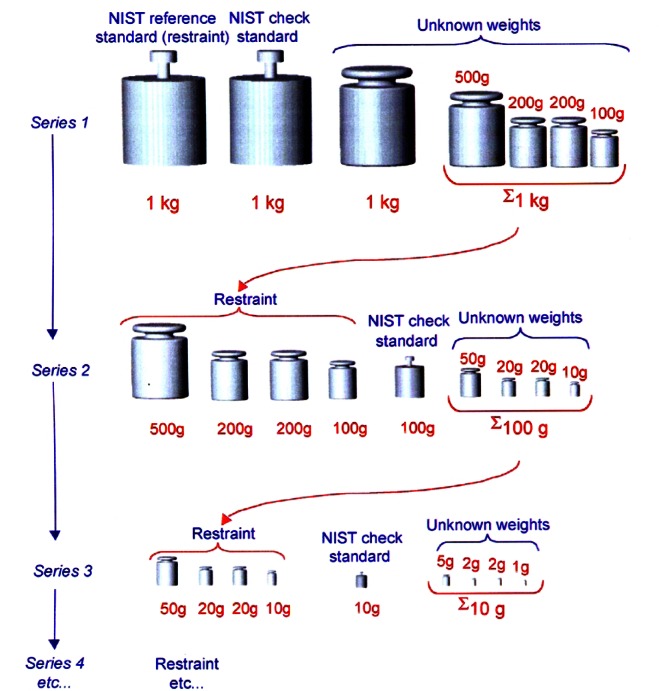
A schematic description of the weighing designs used in the dissemination to submultiples of the kilogram.

Two sets of stainless steel working standards at the kilogram level are used to disseminate the unit of mass to multiples and submultiples of the kilogram. These standards have similar properties as the secondary standards. At NIST, mass measurements traceable to the national standard of mass are regularly performed in the range from 1 mg to 27 200 kg. Typically, weights come in sets consisting of weights of various denominations. For example, a 1 g to 1 kg set consists of the following weights: 1 kg, 500 g, two 200 g, 100 g, 50 g, two 20 g, 10 g, 5 g, two 2 g, and 1 g. Weighing designs were developed to allow one to transfer the unit of mass from the kilogram to other denominations while optimizing the number of measurements and the statistical uncertainty. The protocol used for the calibration of such a weight set is illustrated in Fig. 3. Starting with the first series, four weights of nominal mass of 1 kg are used: (1) 1 kg NIST reference standard, (2) 1 kg check standard, (3) 1 kg unknown, (4) a 1 kg unknown sum denoted by ∑1 kg consisting of a combination of 500 g, two 200 g, and 100 g. Six observations are made using the following difference measurements:

**Table:** 

Observation	(1)	(2)	(3)	(4)
	1 kg	1 kg	1 kg	∑1 kg
*Y*_1_	+	−		
*Y*_2_	+		−	
*Y*_3_	+			−
*Y*_4_		+	−	
*Y*_5_		+		−
*Y*_6_			+	−
Restraint	+			

In this case, the restraint is on the reference 1 kg weight in position (1).

The second series consists of difference measurements among 6 weights: (1) 500 g, (2) 200 g, (3) 200 g, (4) 100 g, (5) 100 g, and (6) ∑100 g consisting of a combination of 50 g, two 20 g, and 10 g. In this case, the restraint is placed on the sum of the weights in positions (1) to (4); this summation is known from series 1; weight (5) serves as a check standard while (6) serves as the restraint for the subsequent series. In this case the following difference measurements are performed:

**Table:** 

Observation	(1)	(2)	(3)	(4)	(5)	(6)
	500 g	200 g	200 g	100 g	100 g	∑100 g
*Y*_1_	+	−	−	−	−	+
*Y*_2_	+	−	−	−	+	−
*Y*_3_	+	−	−	+	−	−
*Y*_4_	+	−		−	−	−
*Y*_5_	+		−	−	−	−
*Y*_6_		+	−	+	−	
*Y*_7_		+	−	−		+
*Y*_8_		+	−		+	−
Restraint	+	+	+	+		

A complete description of the weighing designs for the calibration of mass standards can be found in Ref. [15]. NIST check standards are incorporated into each series of measurements, and a NIST reference standard is only used at the starting series; the tie to the subsequent series is provided by the measurement of the unknowns as determined from the previous series. Similar procedures are used for calibration of the multiples of the kilogram. The observations are corrected for air buoyancy as well as temperature before the masses are calculated using the least squares method [15].

**Fig. 4:**
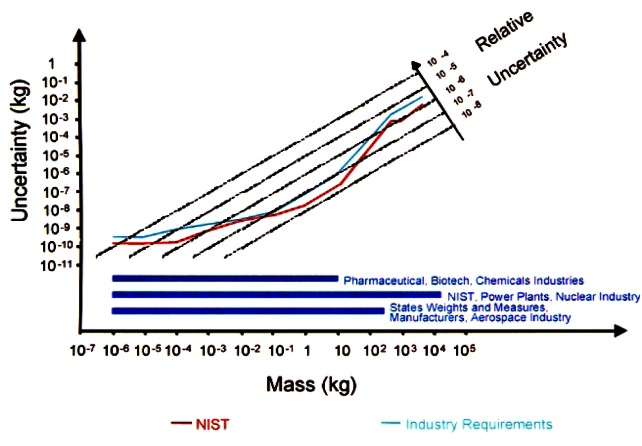
Standard uncertainty of mass calibrations at NIST. Also plotted on this graph is the estimated industry requirement in mass metrology.

Since most mass standards in use are made of stainless steel of similar density to that of the NIST working and check standards, the uncertainty in the buoyancy correction is negligible. Therefore the major contributions to the uncertainty are (1) the uncertainty in the reference standard and (2) the combined repeatability and reproducibility of the balance. Uncertainties due to the volumes of the unknowns cannot be included due to the correlation between the measurements at the time of calibration and at the future time of use of the standard [19]; this component can be added later by the customer based on the value for the air density at the time of calibration of the weight at NIST and at time of use by the customer. Figure 4 shows the combined standard uncertainties and relative combined standard uncertainties plotted against mass values in the most commonly used range from 1 mg to 5000 kg. The “V-shaped” curve is a characteristic of a mass calibration uncertainty curve since the smallest uncertainty is at the 1 kg level where the unit is defined and the uncertainty increases as the unit is disseminated to multiples and submultiples of the kilogram. The curve representing the estimated industrial needs is derived from the tightest requirements in legal metrology and contacts with customers.

### 1.4 Statistical Process Control

Statistical process control procedures are incorporated into the measurements to monitor the precision and accuracy of the calibration process and form the basis of the measurement assurance program for mass calibrations. Measurement assurance programs have been pioneered at NIST since the 1960s [20] with some concepts, such as check standards, dating back to the earlier days of NBS in 1926 [21]. Such procedures have been applied to mass calibrations since 1979 [15]. Only a brief summary is given below.

For each measurement series, the standard deviation of the least-squares fit to the data is calculated and compared to the accepted standard deviation of the balance using F-test statistics [15]. The accepted standard deviation of a balance is the pooled standard deviation based on a very large number of measurements collected over a long period of time. By monitoring the scatter of the data obtained in the weighing design measurements, the F-test monitors the precision of the measurement process. The validity of the F-test relies on the assumption that the scatter of the data is typical of the scatter obtained from previous measurements using the same balance. Control charts are maintained for all the balances used in the calibration services. For each series of measurements, the standard deviation is calculated and compared to the accepted value that is normally obtained from a pooled standard deviation of multiple measurements. Such control charts monitor the performance of the balance; for example, a continuously increasing standard deviation indicates a possible degradation of the balance.

Check standards are mass standards with known or “accepted” mass values. Check standards are incorporated into weighing designs; they are treated as unknowns and their masses are measured and compared to accepted values using T-test statistics. Monitoring the measured mass of an artifact of known mass monitors the accuracy of the measurement process. The validity of the T-test is based on the assumption that the mass of the check standard does not change from its accepted value.

Accepted values for the standard deviations of the balances and the check standards are obtained from yearly updates of control charts. More frequent updates are performed if judged necessary from any unusual results. A control chart for a particular check standard consists of the measured values as a function of time with a computed accepted value and statistical control limits. Control charts monitor the stability and/or drift of mass standards as well as abrupt changes that would indicate possible damage. Such control charts are maintained for check standards covering the full mass scale covered by the calibration services.

### 1.5 Facilities

Electronic mass comparators, fully and partially automated, are used for calibrations in the range from 1 mg to 10 kg, while mechanical balances are used to cover the range between 10 kg and 27 200 kg. Partial automation refers to the automation of the data collection from the comparators and from the transducers monitoring the environment, as well as the automatic analysis of the collected data; full automation also includes the remote operation of the comparators [22]. The environmental conditions in the calibration laboratories are such that the relative humidity is set between 40% and 50% with variations of no more than 5 % per 24 h and the temperature is set between 20 °C and 22 °C with maximum variations of 0.5 °C over a period of 12 h. Electrostatic filters are used to insure proper cleanliness with 97 % filtration efficiency.

A special area is dedicated to the calibration of large weights between 30 kg and 27 200 kg using mechanical balances. The temperature is maintained between 21 °C and 23 °C with maximum variations of 1.5 °C per 12 h. This special area was designed to allow for the receiving, handling, and shipping of large weights and lacks any humidity control.

A clean room facility with tight environmental control houses a state-of-the-art, fully automated and remotely operated 1 kg, 100 g, and 10 kg comparators. The environmental conditions are such that temperature is controlled to within 0.1 °C at a temperature between 20 °C and 22 °C and the temperature gradients are less than 0.1 °C over an elevation of 1 m. The relative humidity is controlled to within 2 % at a relative humidity between 45 % and 50 %. Cleanliness of class 1000 is accomplished with a HEPA filtration system with 99.99 % efficiency for particles of size 0.5 μm or larger.

NIST also maintains facilities for hydrostatic [10,11] and immersed [9] solid density measurements and for the characterization of the magnetic properties of mass standards [23].

### 1.6 Alternative Materials for Mass Standards

Efforts are currently underway to develop and manufacture alternative mass standards to minimize the uncertainty due to the buoyancy correction, the major contribution to the uncertainty. Two of the methods for minimizing this uncertainty are to minimize the difference in volume between the mass standard and unknown or perform measurements in vacuum. Since the behavior of mass standards under vacuum is not yet fully understood and is not practical as a dissemination method, methods to minimize the difference in volume have been investigated. This requires using a material whose density is close to that of platinum-iridium. Tungsten with a density of 19 300 kg/m^3^ satisfies this criteria and reduces the uncertainty associated with the air buoyancy correction to ≈1 μg. At the time of publication of this paper, the possibility of machining a surface of tungsten to an average surface roughness of 100 nm using chemo-mechanical polishing techniques have been demonstrated [24]. Stability tests of such artifacts is planned for the near future.

### 1.7 Characterization of the Surfaces of Mass Standards

In an effort to understand the stability of mass standards, we have characterized the surface roughness and profiles of our national prototype kilograms K4 and K79 using noncontact surface profiling and optical microscopy techniques. K4 and K79 are representatives of the two existing types of surface finish for primary platinum-iridium kilograms. K4 is one of the first 40 replicas made; it was hand polished. K79 is representative of the newer family of Pt-Ir kilograms manufactured at the BIPM turned using a diamond tool. A summary of the results is provided here. For a more detailed account of the work, see Ref. [25].

#### 1.7.1 K4

K4 is one of two mass standards originally allocated to the United States. The second mass standard, K20, is the national standard of mass in the United States. Both K4 and K20 belong to the original group of 40 prototype kilograms. All 40 kilograms were manufactured from the same alloy and by the same process. It is believed that the surface of K4 is representative of the surfaces of the original national mass standards.

**Fig. 5:**
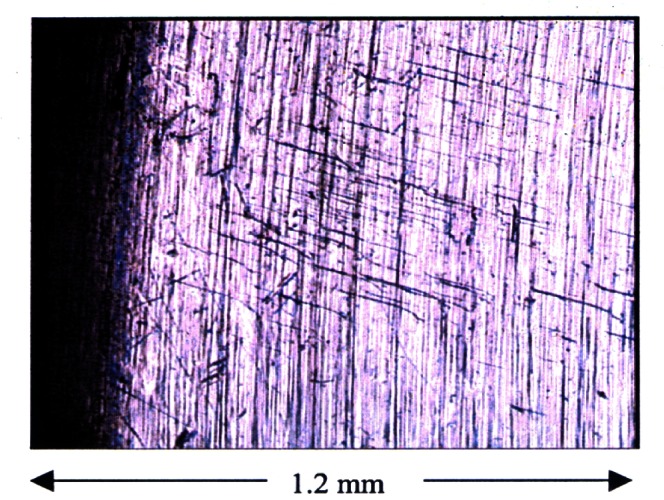
Optical microscopy profile of the bottom surface of K4 near the edge revealing machining lines and wear marks.

The machining lines on K4 are visible to the naked eye. In addition, a few scratches are notably present on the flat and cylindrical surfaces and have been historically reported [19]. Optical microscope profiles reveal, in addition to the machining lines and numerous random scratches, wear lines due to usage on balance pans for a period spanning over more than a century. These lines can be seen in Fig. 5 as short line-segments perpendicular to the machining lines. Using a white-light scanning interferometer, we have measured average roughness values, *R*_a_, ranging from 63 nm to 84 nm at different locations on the flat surfaces of K4 excluding the center. The repeatability in a single measurement location of the average roughness is 1 nm. A detailed mapping of the surface of K4 can be found in Ref. [25].

It is worth noting that in spite of the peculiar surface texture that K4 exhibits, its mass, relative to IPK, has only changed by 41 μg between calibrations at the BIPM in 1889 and 1999. We are currently in the process of reexamining the surface of K4 after cleaning at the BIPM with the hope of shedding some light on the effects of cleaning on surface characteristics and possibly finding at least a qualitative correlation between changes in surface characteristics and changes in mass for platinum-iridium standards.

#### 1.7.2 K79

K79 was acquired by NIST in 1996. It was manufactured at the BIPM in 1986 by turning with a diamond tool. To the naked eye, the surface of K79 looks very specular in comparison with K4. When K79 was placed under the microscope, the improved surface quality was obvious, yet, some peculiarities were found.

The surface roughness was measured with a phase-measuring microinterferometer. The average roughness, *R*_a_, ranged from 10 nm to 15 nm at different locations on the flat surfaces of K79 with repeatability of 1 nm for a single measurement location.

**Fig. 6:**
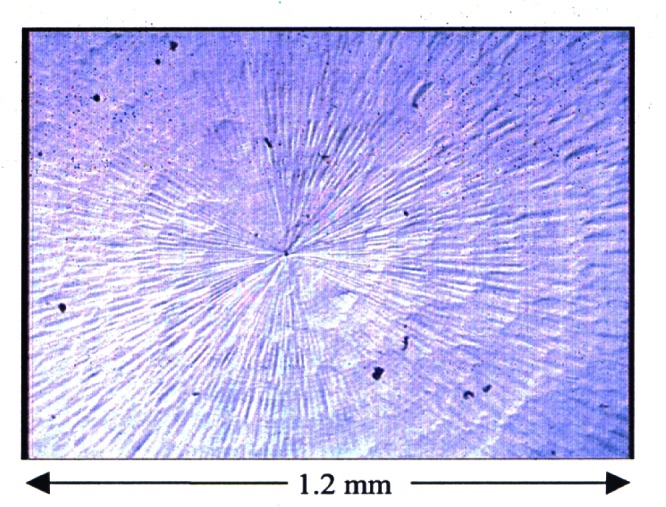
Optical microscopy profile of K79 at the center showing the non-uniform grain size distribution.

In addition, the optical microscopy profiles show evidence of increasing grain size with increasing distance from the center, as shown in Fig. 6. The origin of this nonuniformity in grain size is still under investigation and is most likely attributed to the interaction between the platinum-iridium artifact and the diamond tool or to Pt-Ir material properties. Only a few wear marks were observed compared to the surface of K4.

While it is commonly believed that the prototype kilograms with improved surface properties obtained from diamond turning are more stable than the ones hand polished, long-term history is not yet available to support this hypothesis.

### 1.8 Current Efforts for an Alternative Definition of the Unit of Mass

Efforts to replace the artifact kilogram definition with one based on an invariant of nature have been ongoing for years and have been a challenge to the scientific community. These efforts are based on two approaches: mechanical electrical measurements, and atom counting.

The mechanical electrical measurement approach, which uses what has become known as a “moving-coil watt balance,” is described in detail in this issue by Elmquist et al [26]. The main concept is to compare a power measured mechanically in terms of the kilogram, meter, and second to the same power measured electrically using the Josephson and quantum Hall effects. This links the kilogram to one of nature’s time invariants, the Planck constant *h*. One can thus consider defining the kilogram in such a way as to fix the value of *h* and to use a watt balance to implement the definition and to directly calibrate standards of mass.

The atom counting approach aims at relating the mass of an atom to the kilogram. Within this framework, two paths can be taken:

- Count the number of atoms in a macroscopic object of known mass. This is the basis of the “silicon” project [27]. The main concept is to relate the mass and volume of a 1 kg single crystal sphere of silicon, lattice spacing of a unit cell of the silicon crystal, mean molar mass of the silicon atoms in the sphere, number of atoms in a unit cell, and the Avogadro constant. This approach determines the Avogadro constant and hence the mass of the carbon 12 atom in kilograms.
- Buildup a macroscopic object atom by atom while counting the number of atoms as they accumulate. In one approach currently being pursued, gold ions from an ion beam are deposited on a target [28]. When the total current is measured in terms of the Josephson and quantum Hall effects, and the target is weighed, the result is a value of the Avogadro constant and again the mass of the carbon 12 atom in kilograms.

None of these approaches has been able to rival the present artifact definition yet. However, competing with the present definition requires achieving a minimum level of precision on the order of 1×10^−8^.

### 1.9 Conclusions

The instability and the continuous risks associated with the artifact definition have far reaching consequences. Any change in the kilogram directly affects other related base units, fundamental constants, and derived units such as density, force, and pressure. While the ultimate goal remains to replace the artifact definition with an invariant definition, a goal that is hopefully no longer far out of reach, artifact metrology remains an integral part of mass metrology. Understanding the stability of the artifact definition will, for the near future, remain a crucial factor since no matter how the unit will be realized in the future, the dissemination system will most likely rely on artifacts.

## 2. Force Metrology

### 2.1 The Unit of Force

The General Conference on Weights and Measures (CGPM) ruled in 1901 that force is derived from the basic units of mass, length, and time. In 1960, the 11th CGPM adopted the newton as the unit of force in the International System of Units (SI), where one newton is the force required to accelerate a mass of one kilogram to one m/s^2^, expressed in terms of SI base units as kg · m · s^−2^. At a given location, the force exerted by an object on its supporting structure can be computed from the mass of the object and the free fall acceleration of gravity provided that there are no other vertical forces acting on the object.

Although force is a derived unit, it is of such importance that almost all of the national measurement institutes (NMIs) of the countries participating in the Treaty of the Meter maintain facilities for its realization and dissemination. Indeed, accurate force measurements are required in almost all industries. For example, such measurements are critical when testing mechanical structures such as bridges, buildings, aircraft, and medical prosthetics. Force measurements are required to calibrate the testing machines used to evaluate the strength of materials, to assure quality control in production lines, to measure the thrust of engines, and to certify load cells used in weighing systems.

### 2.2 Force Realization at NIST

Over the range of 44 N to 4.448 MN, NIST realizes discrete static forces by suspending weights of known mass in a known gravity field. In addition, a hydraulic machine capable of generating forces up to 53 MN is available for calibrating large capacity force transducers through comparison with secondary force transfer standards maintained by NIST.

#### 2.2.1 The NIST Deadweight Machines

**Fig. 7:**
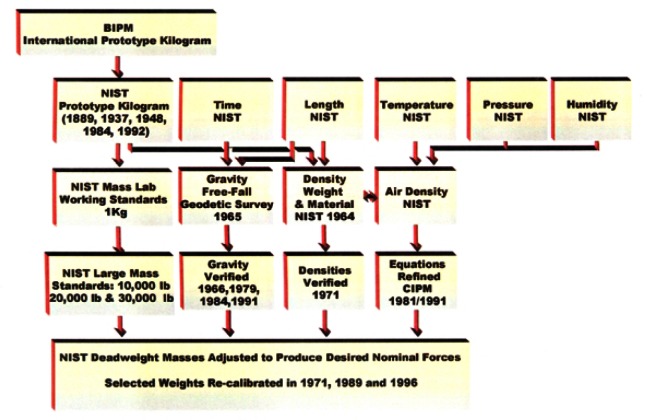
Traceability of NIST primary force standards to fundamental units.

To cover the range of 44 N to 4.448 MN, NIST developed six deadweight machines in which discrete forces are generated by deadweights. The characteristics of these deadweight machines are given in Table 2. The traceability of the primary force standards at NIST to the fundamental SI units is shown in Fig. 7.

**Fig. 8:**
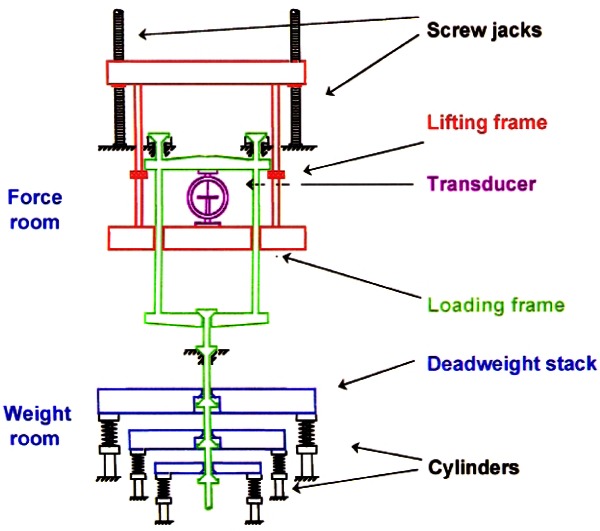
Design principles of the 2.2 kN, 27 kN, and 113 kN deadweight machines.

**Fig. 9:**
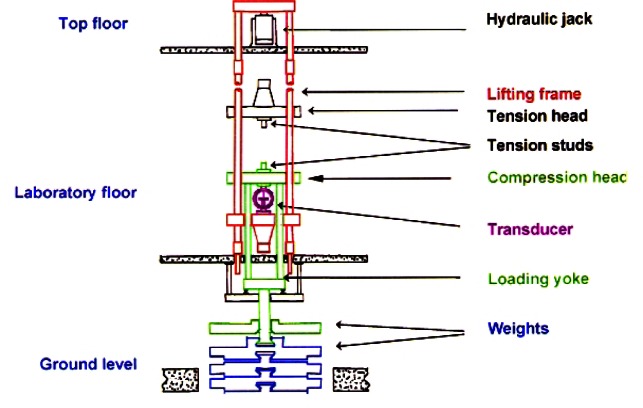
Design principles of the 498 kN, 1334 kN, and the 4448 kN deadweight machines.

The deadweights of all NIST deadweight machines are made of stainless steel. This material was chosen because of its well-known long-term stability. Moreover, the working mass standards used in the NIST Mass Laboratories to calibrate deadweights are also made of stainless steel. Therefore, the transfer errors associated with air buoyancy adjustments are minimized. The particular alloy used for each deadweight machine is listed in Table 2. The design principle involved in the three smallest and the larger NIST deadweight machines are shown in Figs. 8 and 9, respectively.

With the exception of the 27 kN (6.1 klbf) machine, the NIST deadweight machines are fully automated. Further, except for the 27 kN and the 4.448 MN machines, all are equipped with environmental chambers to allow for the characterization of load cells as a function of temperature in a range of −10 °C to 40 °C. Today all NIST deadweight machines are able to apply forces in ascending and descending fashion. Originally, actuation of the deadweights of the 113 kN and 2.2 kN deadweight machines was such that the weight frame needed to be unloaded from the device under test, permitting only return-to-zero loading sequences [29]. During the automation of the force laboratory in 1989, this limitation was overcome by installing pneumatically operated stabilizing mechanisms on these two machines, enabling their deadweights to be changed while the frame is loaded without incurring either excessive wear on the deadweight seats or swinging of the weight frame. These mechanisms retract from the weight frame shafts after each deadweight change. Ascending and descending force sequences can now be applied in these machines. The automation of the NIST deadweight machines has been fully described in Ref. [30].

##### 2.2 kN (505 lbf) Deadweight Machine

Air-powered cylinders manipulate lifting bars that allow the individual deadweights to be applied or removed from the main shaft of the machine at any time during the measurement.

##### 27 kN (6.1 klbf) Deadweight Machine

Hydraulic cylinders raise and lower the deadweights individually onto the main shaft, usually only while the machine is in the unloaded position. When the required deadweight complement is selected, the main shaft is positioned to allow force application to the unit-under-test. Limited ascending and descending loading is possible in this machine under special circumstances. A unique feature of this deadweight machine is that nominal metric forces can be applied by activating an auxiliary deadweight set. This deadweight machine is operated manually.

##### 113 kN (25.3 klbf) Deadweight Machine

Each deadweight is positioned by a pair of hydraulic cylinders. These cylinders allow application or removal of the deadweight to the main shaft at any time. A manually placed set of auxiliary metric conversion deadweights is available for this machine, which produces nominal forces in 4.903 kN increments up to 107.873 kN. These conversion deadweights are used only in nonautomated measurements.

##### 498 kN (112 klbf) Deadweight Machine

Calibration forces are generated in this machine by serially applying deadweights from two different stacks. The minimum force is 13.3 kN (3000 lbf) which consists of the calibrated frame and main shaft of the machine and is always included as the first applied force. All other applied forces must be added to this minimum. The main stack consists of ten 44.4 kN (10 000 lbf) deadweights. The second stack consists of nine 4.44 kN (1000 lbf) deadweights. The deadweights are removed or added to the minimum 13.3 kN (3000 lbf) frame in increments of 4.44 kN (1 000 lbf). An examination of the available deadweight combinations reveals that in some cases it is necessary to unload part of the small stack in order to reach a particular ascending force without first overshooting it.

##### 1.33 MN (300 000 lbf) Deadweight Machine

All deadweights in this machine are applied sequentially with no further individual manipulation possible. The deadweights are of three different sizes. There are thirteen 44 kN (10 klbf) deadweights, four 89 kN (20 klbf) deadweights and three 133 kN (30 klbf) deadweights. This arrangement allows the sequential calibration in ten equally spaced increments of nominal 444 kN (100 klbf), 890 kN (200 klbf), and 1.33 MN (300 klbf) force transducers.

##### 4.45 MN (1 000 000 lbf) Deadweight Machine

This deadweight machine simply applies twenty 222 kN (50 000 lbf) forces sequentially. The main lifting frame raises hydraulically to pick up additional deadweights in the stack. This machine has been fully automated.

#### 2.2.2 Weight Adjustment

When the force laboratory was built in 1965 the force measurement unit in English speaking countries was the pound force (lbf). Accordingly, in 1965, a decision was made to adjust the mass of the weights of the deadweight machines to exert nominal pound forces; the standard pound force being defined as the force acting on a one-pound mass in a gravitational field for which the acceleration of free fall is 9.80665 m/s^2^. The actual mass required to produce a nominal force was computed from the following equation:

$$F=\frac{mg}{9.80665\;{m/s}^{2}}\left(1-\frac{\rho_{a}}{\rho_{w}}\right),$$ (9)

where *F* is the generated standard pound force, *m* is the mass of the weight in lb, g is the local acceleration of free fall at the elevation of the center of gravity of the weight in *m*/s^2^, *ρ*_a_ is the air density, and *ρ*_w_ is the density of the weight material. The uncertainties in the determination of *m*, *ρ*_a_, and *g* are the principal sources of uncertainty in the realized force.

The mass of each weight of the NIST deadweight machines was determined in the Mass Laboratories of the National Bureau of Standards (NBS), the predecessor of NIST. These calibrations were performed in 1965 prior to the assembly of the deadweights in the machines. Over the years some of the deadweights were recalibrated in the Mass Laboratories.

The 498 kN deadweight machine was partially disassembled in 1971, and again in 1979 and in 1989, with most of its deadweights removed and recalibrated each time. Any changes in the mass of the deadweights of the small and large weight stacks were well within the assigned uncertainties. The 2.2 kN deadweight machine was completely refurbished in 1996, and all of its deadweights were removed and recalibrated at that time; the changes in the mass of the weights were again well within the stated uncertainties. The results of the recalibration of the weights indicate that, as expected, the alloys used in both the smaller and larger NIST deadweight machines are very stable over time.

For each of the larger machines, the value of *g* was estimated at the approximate center of gravity of the major components and at the center of gravity of the deadweight stacks. The gravity reference is located on the concrete slab in Room 129 of the first floor of Building 202 at the NIST site in Gaithersburg, MD, where the deadweight machines are located. A second site located in the basement of the same room, approximately 9 m laterally and 2.2 m below the first site, was chosen to establish a permanent reference point for absolute determination of the acceleration of free fall by gravity meter measurements. The assigned value of *g* at this location is (9.801018±5×10^−6^) m/s^2^, this value is based upon an absolute determination conducted by Tate in 1965. All other gravity values are based upon a gravity gradient of −0.000003 m/s^2^ per meter elevation [31]. Subsequent gravity surveys conducted at several locations within the force laboratory by the National Oceanic and Atmospheric Administration confirmed the results obtained in 1965, and tied the measured values to the National gravity base.

During a year, the air density at the Gaithersburg site may vary over a range of 1.145 kg/m^3^ to 1.226 kg/m^3^. In 1965, when the facility was built, a decision was made to use an average yearly value of air density equal to 1.185 kg/m^3^.

#### 2.2.3 Uncertainty in the Forces Realized by Deadweights

The relative combined standard uncertainties of the forces realized by the deadweight machines over the range of 44 N to 4.448 MN incorporate the uncertainties associated with the determination of the mass of the deadweights, the acceleration due to gravity, and the air density as follows:

- The relative standard uncertainty in the determination of the mass of the deadweights, *u*_wa_ <0.0003 %.
- The maximum uncertainty caused by the use of an average air density. This is the largest systematic uncertainty in the applied force and is equal to 0.0005 %. The estimated relative standard uncertainty, assuming a rectangular probability distribution, is *u*_wb_ ≈ 0.0003 %.
- The relative standard uncertainty associated with the variation in the acceleration of free fall with height, assuming a rectangular probability distribution, is *u*_wc_ ≤0.0001 %.

The combined standard uncertainty in the force realized by deadweight application is computed as

$$u_{w}=\sqrt{u_{\text{wa}}^{2}+u_{\text{wb}}^{2}+u_{\text{wc}}^{2}}.$$ (10)

using the values listed in (a), (b), and (c) above yields a combined relative standard uncertainty in the realized force *u*_w_ = 0.0005 % [32].

### 2.3 Comparison Force Calibration

**Fig. 10:**
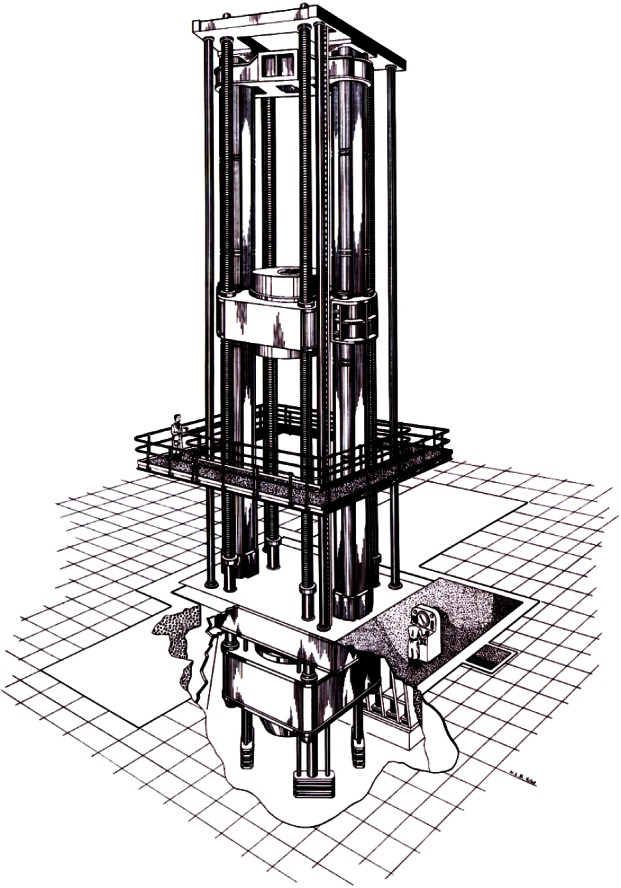
Schematic diagram of the universal testing machine used to perform compression calibrations in the range of 4.5 MN to 53 MN.

**Fig. 11:**
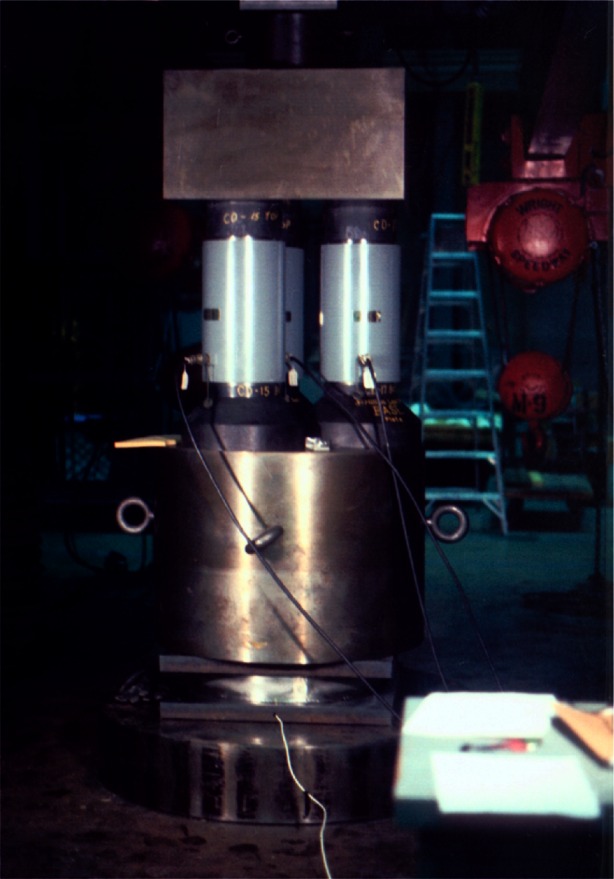
Set up for the comparison calibration of a 13 MN force transducer.

Above 4.448 MN, NIST provides compression calibrations up to 53 MN by comparison with NIST transfer standard strain gage load cells using a 53 MN capacity universal testing machine shown schematically in Fig. 10 [33]. For this purpose, NIST maintains a set of three 4.448 MN NIST transfer standards, each calibrated in the 4.448 MN deadweight machine, and a set of four 13 MN transfer standards each calibrated by comparison with three 4.448 MN transfer standards. In the range of 4.5 MN to 13 MN, three 4.448 MN transfer standards loaded in parallel are used, as shown in Fig. 11. The resulting standard uncertainty, computed by combining in quadrature the uncertainties contributed by each of the three transfer standards, is estimated at 1.7 kN, constant over the interval. Thus, the relative standard uncertainty ranges from 0.038 % at 4.5 MN to 0.013 % at 13 MN. From 13 MN to 40 MN, three 13 MN transfer standards are used. The resulting standard uncertainty is estimated at 5 kN, constant over the interval, with relative standard uncertainties ranging from 0.038 % at 13 MN to 0.013 % at 40 MN. From 40 MN to 53 MN, four 13 MN transfer standards are used resulting in an estimated standard uncertainty of 5.9 kN, and a relative standard uncertainty ranging from 0.015 % at 40 MN to 0.011 % at 53 MN.

**Fig. 12:**
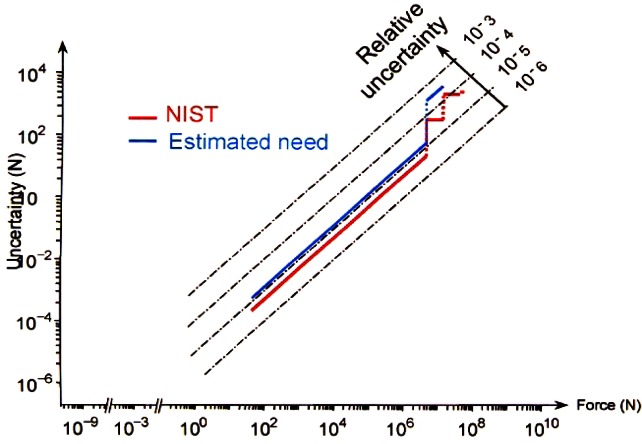
Standard uncertainty in the forces realized at NIST.

The standard uncertainty, in both absolute and relative terms, in the forces realized at NIST over the entire range of 44 N to 53 MN is shown in Fig. 12.

### 2.4 Instrumentation

#### 2.4.1 Deadweight Machine Control Instrumentation

As mentioned previously, except for the 27 kN deadweight machine, all NIST deadweight machines have been instrumented for automated control. With the exception of the mounting and positioning of the force sensor into the deadweight machine, all machine operations can be done under computer control. Details of the automation have been described elsewhere [30]. A force measurement system has two components: a sensing component normally called a transducer, and an indicating component, called an indicator. For example, if the transducer is a proving ring, the transducer’s response, that is the change in diameter as the ring distorts under an applied force, is indicated by a vibrating reed and a spherical button mounted on the end of a micrometer. For strain gage load cells, the change in strain along the surface of the sensing element is indicated by a change in the output signal relative to the voltage applied to the load cell bridge. Only the reading of load cell indicators has been automated. Accordingly, measurements on proving rings are performed manually while measurements of most load cells are performed automatically.

The benefits derived from the automation implemented in the Force Laboratories are numerous. They include the ability to perform measurements with complex loading sequences, precise control of the loading time intervals, and more consistent indicator readings. In addition, evaluations of prototype load cells involve the determination of the effects of environmental factors on load cell characteristics. For some of these tests positioning of the load cell in the deadweight machine is required only once, at the beginning of a test. The associated equipment required for these environmental tests has also been automated. Thus, the thermal bath units used to heat and cool the environmental chamber, and the sensors used to monitor conditions within the chamber, including the temperature of the load cell, are also under computer control. These tests, which typically take several days, can thus be conducted around the clock without any manual intervention.

#### 2.4.2 Voltage Ratio Instrumentation

The force applied to a load cell produces a change in the resistive unbalance in the load cell strain gage bridge. For most load cell measurements performed at NIST, this resistive bridge unbalance is measured with a calibrated NIST voltage-ratio indicating system. The NIST indicating system supplies direct current excitation to the load cell, through the use of a specially built power supply which applies DC voltages to the load cell excitation input leads of ±5 V relative to the load cell ground wire, yielding a 10 V difference between the leads. This excitation voltage is stable to within ±5 mV over a time period of 15 s. The power supply was designed to switch internally the wires going to the load cell terminals by means of a computer command, thus reversing the polarity of the excitation signal to the load cell. This action makes it possible to cancel out small thermal biases in the strain-gage bridge and connecting wires, as well as any zero offsets in the rest of the indicating system. The switching is not done if the load cell is not designed to accommodate reversed polarity excitation. The excitation voltage and the load cell output voltage are sampled simultaneously by an 8.5 digit computing voltmeter operating in the voltage-ratio mode; the voltmeter calculates the corresponding voltage ratio internally and returns that value in digital form to the computer. The voltmeter is read several times, with the excitation voltage polarity reversed between readings; the final voltage ratio is taken as the average of the voltage ratios measured at each polarity. The sampling time at each polarity, and the delay after switching polarity before resuming the sampling, are specified by the operator through the computer control/acquisition program. A typical time for one complete voltage ratio reading is 10 s. This time can be shortened or lengthened as appropriate for the measurement being conducted. Calibration of the voltmeters in the voltage-ratio mode is done by providing calibrated DC voltage signals simultaneously to both inputs, with the DC calibrated signals derived from a 10 V Josephson junction reference voltage array maintained by the Electricity Division of the NIST Electronics and Electrical Engineering Laboratory. The NIST Electricity Division calibrates the Force Laboratories voltmeters each year. In the Force Laboratories the calibration of all voltmeters is maintained by monthly comparison with the voltmeter most recently calibrated by the Electricity Division. This is accomplished through the use of two devices: a precision voltage reference divider having a 100:1 ratio and a load cell simulator that is stable to within ±5 nV/V over a 24 h time interval.

#### 2.4.3 Uncertainty in Voltage Ratio Measurement

The standard uncertainty associated with the digital voltmeters used in the NIST Force Laboratories for voltage-ratio measurement arises from the following:

- The uncertainty in calibration of the voltage-ratio of the voltmeters as determined by the NIST Electricity Division using a Josephson junction voltage array as a primary standard; the relative standard uncertainty in the voltage ratio over the range from 1 mV/V to 10 mV/V is
  $$u_{\text{va}}\leq 0.0002\%.$$
- Differences between voltmeter calibrations performed by the NIST Electricity Division and comparisons to a 10 mV/V reference ratio obtained with a precision reference divider used in the Force Laboratories to track the voltmeter drift. The estimated relative standard uncertainty of these differences is *u*_vb_ ≈ 0.0003 %.
- The repeatability in measurements for each voltmeter (made at one-month intervals) of the 10 mV/V response relative to the precision reference divider; the relative standard uncertainty for an individual voltmeter is *u*_vc_ = 0.0003 % of the reference ratio.
- The non-linearity in the voltage-ratio measurement response of the voltmeters in the range of 1 mV/V to 10 mV/V; the estimated relative standard uncertainty based on Electricity Division data is *u*_vd_ ≈ 0.0001 % of the reference ratio.

The combined standard uncertainty in the voltage-ratio instrument is given by:

$$u_{v}=\sqrt{u_{\text{va}}^{2}+u_{\text{vb}}^{2}+u_{\text{vc}}^{2}+u_{\text{vd}}^{2}}.$$ (11)

Inserting the values given above yields a relative standard uncertainty for the voltage ratio of about 0.0005 %.

### 2.5 Procedures

The forces realized at NIST are disseminated to industry, government, and the research community through the force calibration services that NIST provides. The objective in calibrating a force sensor is to determine the functional relationship between the applied load and the sensor response. In the Force Laboratory, this is accomplished by applying a series of well-known forces to the sensor and observing its response on a readout instrument. Many force sensors can be calibrated in both tension and compression modes with the responses expected to be somewhat different in each mode. Due to hysteresis effects, the response may also depend on whether the loads are applied in ascending or descending order. Accordingly, for any one sensor, there may be several distinct calibration curves.

Force calibrations at NIST are usually performed according to the procedures specified by the American Society for Testing and Materials (ASTM) Standard Practice E74 [34]. A minimum of 30 forces are applied during the course of each calibration. These forces are applied in two or more calibration runs with typically three positions of the sensor in the deadweight machine to minimize the machine-sensor interactions [35–36]. The applied forces are selected at approximately every 10 % increment over of the entire calibration range. Upon request, a device may be calibrated by modified procedures tailored to meet particular end uses. For example, additional loads may be added, and the loading sequence may include both ascending and descending loads to thoroughly characterize the hysteresis of the force transducer. To obtain the actual response of the transducer, the indicator reading observed during a force application is corrected for the reading observed without any force application. The calibration curve is derived by fitting a polynomial to the data using the method of least squares. The calibration curve is of the form:

$$D=A_{0}+\sum A_{i}F^{i},$$ (12)

where *D* is the response, *F* is the applied force, *A_i_* are the coefficients yielded by the least-squares fit and the summation is usually carried to an order of two or three.

ASTM E 74–95 [34] specifies a standard deviation that is calculated from the differences between the values observed during the course of calibration and the corresponding values computed from the calibration curve. This standard deviation is given by:

$$s=\sqrt{\frac{\sum d_{j}^{2}}{\left(n-m\right)}},$$ (13)

where *s* is the standard deviation, the *d_j_* are the differences between the measured and calculated deflections, *n* is the number of measured deflections, and *m* is the number of degrees of freedom in the polynomial, which is the degree of the polynomial plus one. This standard deviation is one of the terms used in estimating the combined uncertainty as reported in the NIST calibration reports where it is denoted as *u*_r_. The uncertainties contained in *u*_r_ are ordinarily much greater than the uncertainty in the applied load. The two major sources of systematic errors are mechanical misalignment and load-time effects [35,36]. Complex mechanical interactions between the force sensor and the deadweight machine can cause bending, shear, and torsional loads to act in combination with the precisely known vertical force. In addition, the transducer response is also dependent upon the load history. A detailed statistical analysis that yields separate estimates of uncertainty arising from various possible sources of error can be found in Ref. [37].

The combined standard uncertainty stated in NIST force calibration reports is computed using the following equation:

$$U_{c}=\sqrt{u_{w}^{2}+u_{v}^{2}+u_{r}^{2}}.$$ (14)

where *U*_c_ is the combined standard uncertainty as defined in Ref. [17], *u*_w_ is the standard uncertainty of the applied deadweight, *u*_v_ is the standard uncertainty of the calibration of the voltage-ratio measurement instrumentation, and *u*_r_ is the standard deviation calculated accordingly to ASTM E 74–95. It should be noted that the term *u*_v_ applies only in calibrations involving voltage-ratio measurements performed using the NIST voltmeters.

In addition to performing calibrations, the Force Laboratory performs pattern evaluation tests of load cells used in weighing systems, which provide the basis for the classification by weights and measures officials of load cell families used in weighing systems. These tests are performed in accordance with the specifications of the National Conference of Weights and Measures Publication 14 [38], and a similar international standard, OIML R60 [39], adopted by the International Organization of Legal Metrology. While there are some differences between the national and international standards, they are minimal. Both procedures prescribe deadweight loading tests of prototype load cells for the linearity, hysteresis, repeatability, and creep over a temperature range of −10 °C to 40 °C. In addition, both require that canister load cells be tested for atmospheric pressure sensitivity over a range of 95 kPa to 105 kPa.

### 2.6 Current Force Metrology Research

Two main efforts are now underway at NIST in the area of force metrology. They include:

- The development of a research laboratory for the realization, measurement and repeatable dissemination of very small forces (in the micro- and nano-newton range) to address the emergent force measurement needs of a growing class of nano-technologies, including atomic microscopes, nanoindentors, and micro-electromechanical systems (MEMS); and
- The development of a testing facility to assess the susceptibility of digital load cells to electromagnetic radiation.

## Figures and Tables

**Table 1:** Year of calibration and masses reported by BIPM for the U.S. prototypes

Year	K20	K4
1889	1 kg – 0.039 mg	1 kg – 0.075 mg
1937	1 kg – 0.021 mg	
1948	1 kg – 0.019 mg	
1984	1 kg – 0.022 mg	1 kg – 0.106 mg
1992	1 kg – 0.021 mg	
1999	1 kg – 0.039 mg	1 kg – 0.116 mg

**Table 2:** Characteristics of the six NIST deadweight machines

Capacity, kN (klbf)	2.2 (0.505)	27 (6.1)	113 (25.3)	498 (112)	1334 (300)	4448 (1000)
Min. load,						
kN	0.044	0.44	0.89	13	44	222
(klbf)	(0.01)	(0.1)	(0.2)	(3)	(10)	(50)
Min. increment						
kN	0.022	0.22	0.44	4.4	44	222
(klbf)	(0.005)	(0.05)	(0.1)	(1)	(10)	(50)
Compression setup space:						
Vertical (m)	0.25	0.61	0.76	1.02	1.65	1.98
Horizontal (m)	0.29	0.47	0.50	0.71	0.91	0.86
Tension setup space:						
Vertical (m)	0.56	0.76	0.91	2.16	2.49	4.45
Horizontal (m)	0.29	0.64	0.66	0.71	0.91	1.17
Alloy of weights						
AISI series	302	302	302	410	410	410
Density of weights at 20 °C						
kg/m^3^	7890	7890	7890	7720	7720	7720
